# ‘She didn't know how to go back’: School attendance problems in the context of the COVID‐19 pandemic—A multiple stakeholder qualitative study with parents and professionals

**DOI:** 10.1111/bjep.12562

**Published:** 2022-11-07

**Authors:** Brontë McDonald, Kathryn J. Lester, Daniel Michelson

**Affiliations:** ^1^ School of Psychology University of Sussex Brighton UK; ^2^ Institute of Psychiatry, Psychology and Neuroscience King's College London London UK

**Keywords:** COVID‐19, mental health, parents, qualitative, school attendance problems

## Abstract

**Background:**

The COVID‐19 pandemic resulted in school closures worldwide and unexcused absences have increased since schools reopened.

**Aims:**

Drawing on multiple stakeholders' perspectives, we aimed to (i) develop a detailed understanding of how school attendance problems (SAPs) have manifested for primary school‐aged children in the context of COVID‐19; and (ii) identify promising community‐based intervention strategies.

**Methods:**

We used a qualitative design with two sequential phases of data collection. Phase 1 involved insight generation using qualitative surveys with parents and professionals working in primary education settings. These results were used to guide in‐depth stakeholder interviews in Phase 2.

**Sample:**

Phase 1 included 29 parents of primary‐school children experiencing SAPs and 19 professionals. Phase 2 included 10 parents and 12 professionals. Parents were recruited through social media; professionals were identified through schools and associated networks in Southern England.

**Results:**

Attendance was particularly challenging for children with special educational needs and pre‐existing anxiety problems. Compounding factors included COVID‐related anxiety, difficulties adapting to new school routines, poor home‐school communication and collaboration, and concerns about academic catch‐up. Effective support was characterized by schools and families working closely together. Recommendations for practice improvements centred on early intervention, re‐building parent‐school relationships, peer support for parents, and improving special educational provision.

**Conclusion:**

New interventions for SAPs must be sensitive to the ongoing COVID‐19 context. Help should be easily accessible in the community and address modifiable risk and protective factors for individual children, in family systems, and at the home‐school interface.

## INTRODUCTION

Poor school attendance is associated with adverse outcomes including low academic attainment, school drop‐out, unemployment and mental disorders (Egger et al., 2003; Kearney & Graczyk, 2014; O'Connor, 2017). School attendance problems (SAPs) typically emerge in primary school (Havik et al., 2014) and there is increasing recognition that intervening early is instrumental in mitigating persistent absences and associated negative outcomes (Cook et al., 2017; Ehrlich et al., 2014).

Previous research has examined a broad range of individual child factors (e.g., mental health, special educational needs [SEN], physical health and sleep problems); parental factors (e.g., parent mental health, school engagement, family functioning and parenting); and environmental factors (e.g., school climate, school and home relationship, community support, socio‐economic resources) as contributors to SAPs (Kearney, 2016). These factors can be understood within a bioecological model, which positions the child in a hierarchy of environments that can influence the onset and maintenance of SAPs over time (Melvin et al., 2019). The majority of the available research has used quantitative methods, with relatively little attention paid to the explanatory models and intervention priorities of children, parents, education staff and other stakeholders (Heyne et al., 2019). As well as a lack of qualitative research on stakeholders' perspectives, the available research is also skewed towards secondary school samples (Cook et al., 2017).

Furthermore, COVID‐19 has caused unprecedented disruption to schooling worldwide. In the UK, both primary and secondary schools were closed to most pupils from March to July 2020 and January to March 2021. SAPs have increased in the aftermath of these closures, with 22% of children being persistently absent in England (i.e., missing over 10% of sessions) in Autumn 2021. Although COVID‐related illness has played a direct part in many absences (i.e., due to self−/family isolation and the effects of long COVID), there is also evidence to suggest the influence of psychological and contextual factors (Children's Commissioner, 2022). When COVID‐19‐related absences were removed from absence statistics, 12% of children were persistently absent in the 2021‐/2022 academic year, compared with a pre‐pandemic persistent absence rate of 10.8% (Department of Education, 2022).

Data indicate that child and parental mental health have worsened over the course of the pandemic (Ashikkali et al., 2020; Creswell et al., 2021; Panda et al., 2021), particularly in families with children who have SEN, families living on low incomes, and families with pre‐existing child and parental mental health difficulties. Families have also experienced major disruptions to support services, with one UK study suggesting that social care input had been suspended for 80% of UK families during the first period of COVID‐19 ‘lockdown’ in spring 2020 (Waite et al., 2020). Capacity issues in children's health and social care have persisted throughout the pandemic, as seen in growing waiting lists and staff vacancies (Foster & Foley, 2022). Moreover, the extended use of COVID‐19‐related social distancing restrictions (e.g., suspending parent‐teacher consultations on school premises) has continued to disrupt channels for effective communication between parents and schools, even after schools reopened (Kim et al., 2021).

Existing interventions for SAPs have not been designed or evaluated in the context of COVID‐19. Empirically supported approaches typically target child factors (especially the amelioration of child anxiety problems), while paying relatively little attention to wider risk and protective factors beyond the immediate family system (Cook et al., 2017). As we emerge from the COVID‐19 pandemic, there is a need for more contextually sensitive interventions that do not rely on already over‐stretched specialist mental health and social services for delivery.

The current study explores the nature of SAPs and stakeholders' service priorities in the context of the COVID‐19 pandemic. This is the first step in a larger research programme that aims to develop a novel intervention for families with primary‐school children experiencing SAPs. While much previous research on SAPs has been either survey‐based or limited to single‐stakeholder perspectives (Cunningham et al., 2022; Heyne et al., 2020; Reid, 2016), the current study seeks to assimilate complex real‐world perspectives from multiple stakeholders in southern England.

We investigated the following research questions:

What are the factors associated with SAPs for primary school‐aged children within the context of the COVID‐19 pandemic?
(i) What formal and informal sources of support have been provided to address new/persistent SAPs during and prior to the pandemic, and (ii) to what extent have these approaches been effective?
(i) What other intervention approaches may be helpful for children and families affected by SAPs, and (ii) what are the opportunities and potential strategies for implementing these?

## METHODS

### Study design

We used a qualitative, mixed‐method design with two sequential phases of data collection (Morse, 2010). In Phase 1, a hybrid deductive‐inductive insight‐generating approach was applied through qualitative surveys with parents and professionals (Braun et al., 2020). Findings from Phase 1 were used to guide in‐depth qualitative interviews in Phase 2. Approval was obtained prior to study commencement from a UK Research Ethics Committee (Reference: ER/BM333/6).

### Participants

Eligible participants included parents of a child who was (i) enrolled in a state‐funded mainstream primary school within the local authorities of East Sussex, West Sussex, and Brighton and Hove; and (ii) had experienced SAPs over the academic year 2020–2021. Parents who identified more than one child with SAPs were asked to select the child who had the greatest difficulty attending school. Parents were recruited through targeted advertisements placed on relevant parent and community social media groups across the aforementioned local authorities in England.

We also recruited professional stakeholders working with primary school children in the same localities. Professionals were recruited through contacts with local authorities and educational psychology services, and by directly approaching five schools with the highest persistent absence rates from each district within the relevant local authorities (Department of Education, 2021).

Twenty‐nine parents and 19 professionals completed Phase 1 data collection. The 22 participants in Phase 2 included a sub‐sample of seven participants (six parents; one professional) from Phase 1 who consented to further participation and were available for interviews. An additional 15 participants (four parents; 11 professionals) entered directly into Phase 2.

### Measures

#### Phase 1 surveys

Surveys were tailored for the two main stakeholder groups and consisted of six open‐ended, free‐text questions (see Appendix S1).

For parents, two questions asked about the nature of SAPs experienced (i) before the pandemic (i.e., before national school closures in March 2020); and (ii) during the Autumn term 2020 and Spring term 2021 (corresponding to time periods immediately following national school closures). Additional questions asked parents to describe their own feelings about their child attending school during the pandemic; reasons that might explain their child's difficulties attending school; formal and informal support received and whether it had been helpful or not; and additional support that would be helpful for their family.

The survey for professionals asked about specific high‐risk groups for SAPs before and during the pandemic; specific difficulties faced by families when preparing to return from school closures; explanatory factors for SAPs; examples of local good practice for SAPs; and prioritized areas for service development.

#### Phase 2 interviews

Semi‐structured topic guides were created after a preliminary analysis of survey data. Particular attention was given to links between child anxiety, parenting behaviours and SAPs; sources of informal and community‐based support; school and statutory responses to SAPs; and the quality of home‐school relationships and implications for addressing SAPs (see Appendix S1).

### Procedure

Phase 1 data was collected during the Spring term 2021 (March–May) when children were returning from the second period of extended school closures (January–March 2021). Prospective participants enrolled in the study via a web‐link that was circulated in digital recruitment materials. The link led to two eligibility questions that asked parents if (i) their child had experienced difficulties attending school in‐person during and/or after the summer term of the academic year 2020‐2021 when schools reopened following the first round of national closures; and (ii) whether their child was enrolled in a mainstream state primary, infant or junior school in Sussex. Eligible participants then accessed a detailed participant information sheet and were invited to complete a consent form and standard demographic proforma followed by the relevant qualitative survey questions. Participants received a £5 e‐voucher upon completion and were invited to leave contact details to participate in future studies.

Phase 2 participants were enrolled in two cohorts. The first cohort was comprised of Phase 1 respondents who opted in to receive further research communications. This group received a new web‐link 3 months after completing the initial survey. The link led to a participant information sheet and consent form for the interview stage. The second cohort was comprised of newly recruited participants who entered the study directly into Phase 2 without initially completing a Phase 1 survey. The latter group completed eligibility screening, followed by informed consent and a self‐completed demographic proforma.

Interviews were completed in the Summer term and Summer holidays 2021 (July–August) by telephone (*n* = 7) or via Zoom video‐conferencing software (*n* = 15). Interviews lasted approximately 45–60 mins, were recorded, transcribed verbatim and anonymized. Participants received a £10 e‐voucher.

### Analysis

Thematic framework analysis (Gale et al., 2013) was conducted sequentially on the survey and interview data. A hybrid deductive‐inductive approach was applied to both data sources, using a combination of deductive coding, where an initial coding framework followed logically from the research questions, with refinements made through data‐driven inductive coding. The analysis followed the steps of familiarization (a process of ‘getting to know’ the data extensively with multiple readings of the transcripts); identifying a framework (creating framework categories from research questions and initial readings of the data); indexing (organizing the data into the framework categories while iteratively adapting the framework); charting (organizing the data into a more manageable format); and mapping and interpretation (bringing together themes in the data to map and interpret the data set as a whole) (Ritchie & Lewis, 2003). Analysis moved back and forth through steps when required. A preliminary coding framework was developed by the first author (BM) from predetermined themes informed by the research questions and by data‐driven codes. This coding framework was then applied and continuously revised throughout the survey analysis. Higher‐order themes were refined further after Phase 2 interviews and in consultation with senior authors KL and DM.

The analysis was led by BM who is a PhD researcher based in an academic department of Psychology and with a background in education as a trained primary school teacher. The lead author took a critical realist perspective, which involved interpretation of the data with respect to existing theoretical and empirical accounts of SAPs, while also drawing from their own lived experiences as a teacher (Fletcher, 2016). All authors met weekly or fortnightly throughout data collection and analysis, and discussed assumptions and challenged interpretations in the context of these lived experiences.

## RESULTS

### Participant characteristics

Phase 1 parent participants (*n* = 29) were predominantly mothers (*n* = 25; 86%) and White British (*n* = 26, 89%). Nine (31%) reported a household income below the UK median (<£29,999), seven (24%) reported a household income between £30,000 and £49,999, 11 (38%) reported a household income above £50,000, and two (14%) preferred not to disclose their household income.

Just over one‐third (*n* = 11; 38%) of children were female, and the mean age was 8.3 years (SD = 1.8, range = 5–11). Ten children (35%) were eligible for Free School Meals (i.e., school lunches provided at no cost to families who are in receipt of welfare benefits) compared to the national average of 19%. Fourteen children (48%) had SEN; six (21%) had an Education Health Care Plan (a document which sets out the education, healthcare and social care needs of a child for whom extra support is needed other than which the school can provide directly) and eight (69%) had additional learning needs such as identified autism or dyslexia but were awaiting assessment for an Education Health Care Plan. Based on parent report, 10 children (35%) met the Department for Education's (Department for Education, 2022) national criteria for ‘persistent absence’ in Autumn term 2020 (absent from >10% of total sessions); nine children (31%) met this same persistent absence criterion in the Spring term 2021.

The professionals (*n* = 19) included nine headteachers (47%), four Education Mental Health Practitioners (EMHPs; 21%), two educational psychologists (11%), two class teachers (11%), and two SEN coordinators (SENCOs; 11%).

Phase 2 parent participants (*n* = 10) were all White British mothers. Six (60%) reported a household income below the UK median, one (10%) reported a household income between £30,000 and £49,999, and three (30%) reported a household income above £50,000. Exactly half of the children were female, and the mean age was 8.4 years (SD = 2.0, range = 5–11). Three children were eligible for Free School Meals (30%), four (40%) had SEN, one (10%) had an Education Health Care Plan, and three (30%) had additional learning needs. Set against Department for Education criteria, 60% of the children were persistently absent in Spring term 2021 and 40% in Summer term 2021.

Participating professionals (*n* = 12) included two headteachers (17%); two EMHPs (17%); one educational psychologist (8%); two class teachers (17%); one clinical psychologist (8%); one School Mental Health and Emotional Wellbeing Advisor (8%); and three Education Support, Behaviour, and Attendance Service (ESBAS) officers (23%).

### Thematic framework analysis

Results have been organized around three over‐arching themes: (1) contributors to SAPs; (2) responses to SAPs; and (3) areas for additional support.
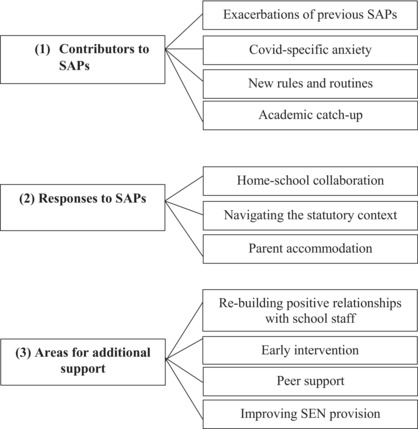

1. Contributors to SAPs

*Exacerbations of previous SAPs* Many parents noted that SAPs were long‐standing but had worsened during the pandemic. This was particularly the case for children with SEN, for whom school was already a challenging context (e.g., due to sensory stimulation, difficulties with social situations and academic demands). Returning to in‐person education after extended periods at home was noted as being particularly anxiety‐provoking for these children, requiring a further period of adjustment.

Obviously with Covid in the way, that obviously impacted it because she's had the time away from school. [Child's name] has always struggled with school, she was diagnosed with autism, PDA [Pathological Demand Avoidance] and anxiety.

Parent survey.

Parents and professionals alike explained that home learning tended to be viewed positively by children with pre‐existing anxiety problems. Like children with SEN, home learning removed anxiety‐inducing situations, such as children with social anxiety being able to avoid social interactions. Renewed challenges were presented when children were faced with a return to in‐person education.

She has had problems in the past with anxiety about school, but since the lockdowns, it has been much worse as she would rather be at home, and home schooling has made her aware that there could be another option. She is hoping for another lockdown!

Parent survey.

Where they [children] may have experienced anxiety, maybe a bit more than other kids before Covid, I think it was just exacerbated, it's like home for them is a safe space, and they spent all that time at home with their family, especially if home is a relatively safe loving place. And the thought of going back into school, it's just like they don't want to do it because they've been a home for so long and it's become comfortable for them.

EMHP Interview.

*COVID‐specific anxiety* A number of parents noted that their children, including those with no pre‐existing anxiety problems, had experienced SAPs because of anxieties specifically related to catching and spreading COVID‐19. Some parents suggested that their child's worries were compounded by the abrupt switch to remote learning in January 2021 following a sudden change in Government policy for English schools. Again, this was especially true for children with SEN, who appeared to have had more difficulties with the changing messages around the safety of schools. Furthermore, some children, particularly in families who had strictly followed guidelines around social distancing, found it difficult to be around large numbers of children when schools re‐opened.

[Child's name] has autism and one of his biggest issues is with anxiety. So when his elder brother's school was shut, the entire school went into complete shut‐down and [child's name] was struggling to comprehend how his school could be classed as safe. Whereas [sibling]'s school is like, ‘No we can't have you here, it's not safe enough.’

Parent interview.

I think, because people have drummed into her so much that you've got to stay away from people to keep your distance … she didn't know how to go back into that environment and interact with other children. She was saying, ‘I don't want to go, I don't want to see my friends. I miss them, but I don't know want to talk to them.’

Parent interview.

Professionals noted that parents' own concerns about COVID‐19 were having an impact on school attendance in some families.

One of the biggest reasons that young people in primary are not attending school is because of parent anxiety about their young person getting Covid and passing it around to them. And you know, they might be living with grandparents, and so on.’

ESBAS Officer interview.

Families have been fearful of sending their child in case they get infected by the virus and spreading it to family members. Some families have commented that they have seen, through social media, how some families have not complied with restrictions, which has increased their fear of sending their children in to mix with the children of these families. Headteacher survey.

*New rules and routines* Changes in the normal operation of schools (e.g., ‘bubbles’ imposed to restrict mixing, frequent teacher/pupil absences due to self‐isolation mandates and prohibitions against parents entering school grounds) were also identified as exacerbating children's anxiety and consequently affecting school attendance. Parents and professionals reported that these changes often felt overwhelming for children, particularly as national and local public health regulations were frequently adjusted.

Some children appear to have experienced anxiety about the new rules in place at school and worry about ‘getting it wrong.’

Educational Psychologist survey.

Parents of children with SEN reported particular difficulties because many resources and supports (e.g., quiet spaces or external professional services) had been restricted by new regulations.

What was happening is he couldn't cope with the rules and there wasn't any actions really made for SEN. He did know there were certain things he wasn't allowed to do but he couldn't cope in the large classrooms in the first place. Even though they're in year group bubble, or class bubble, he could not cope with that, it was too noisy. He had his safe space taken away.

Parent interview.

*Academic catch‐up* Parents and professionals described how some children felt intense pressure to catch up with schoolwork after periods of home learning. This was particularly evident for older primary school children in anticipation of standardized tests (i.e., Standard Assessment Tests, SATs, taking place at the end of primary school).

She puts a lot of pressure on herself to be the best and if she's not the best she gets quite upset. And I think she was worried that she had got behind in lockdown and that other people might have done more, or that she wouldn't pass any tests that she needed to.

Parent interview.

Some professionals postulated that children from low‐income families were more likely to worry about academic impacts because of fewer resources (e.g., books and internet‐enabled devices) to support home learning.

Children who are in receipt of pupil premium or who are low attainers have not necessarily had access to the resources needed for effective home learning, therefore their return to school is stressful for them.

SENCO survey.

2 Responses to SAPs

*Home‐school collaboration* Collaboration between families and school staff (e.g., in the form of regular meetings, part‐time timetables, support for external referrals and proactive advice) was identified by parents as being helpful in managing SAPs. Conversely, parents of children with persistent absences shared experiences of poor collaboration and generally negative relationships with school.

We were really lucky, and probably not all teachers were at the same level. We did have an exceptional teacher and [they] couldn't have done much more to help me personally. I was really pleased and very lucky. I know other parents that have not been so lucky with their schools, so I feel fortunate.

Parent interview.

Parents and school staff diverged on their attributions for poor home‐school collaboration. School staff expressed concern that parents were sometimes not sharing information about their children's difficulties at a sufficiently early stage. Parents, on the other hand, described feeling judged or dismissed by school staff. Relatedly, some professionals observed that schools often formed assumptions about parents being ‘lazy’ or ‘not trying hard enough’ to get their child into school.

Communication is key. We have a lot of parents saying that they feel that schools don't listen to them so they just give up talking. When you break that down and dig deeper, it's sometimes a reluctance on the parent's side to talk to the school because they may feel judged or they may feel embarrassed that they can't get their child to school, especially when they're in primary school.

ESBAS Officer interview.

The school's approach, particularly the headmaster's approach, made it virtually impossible for it to work in any way, shape or form. I think, unfortunately, he decided it was a behaviour issue, which was really unfortunate because it really wasn't at all. It was classic anxiety. His approach and kind of attitude made it [so that] we just didn't have any choice but to take her out of the school. I didn't feel that I was left with any choice, there was nothing that I could actually do, they wouldn't provide any support at all.

Parent interview.

It was also generally acknowledged that COVID‐19 restrictions limited informal communication between school staff and parents. Examples were given of prohibitions against spontaneous meetings on school premises before and after school. The loss of such opportunities was noted as being especially problematic for families with children in their first and second years of primary school, for whom parent‐school relationships were not yet well established at the onset of the pandemic.

*Navigating the statutory context* Some parents expressed strong dissatisfaction with the approaches taken by schools and other authorities when applying statutory requirements for school attendance. Threats of financial fines and prosecution were seen as rigid, insensitive and hasty. Likewise, the majority of professionals felt that punitive measures were not helpful in addressing SAPs, particularly when child and parental mental health problems were implicated in the onset and maintenance of SAPs.

Some teachers use threats of mum and dad being fined money. I've seen that and that totally doesn't work. You can just see it makes the child way more anxious because then they've got this added pressure on them cause they are like, ‘Oh God now mum and dad are going to be in trouble.’ And I don't find that helpful.

EMHP interview.

*Parent accommodation* Respondents offered a diverse range of views about the consequences, both positive and negative, of parents' habitual responses to children's SAPs. Although the term ‘parent accommodation’ was not specifically used, many professionals described parental responses, which fitted a pattern, where parents mitigated their child's distress by facilitating the avoidance of anxiety‐provoking situations. This often involved the parent keeping the child home from school in response to, or in anticipation of, their child's anxiety about attending school. Some parents felt that keeping their child off school was unhelpful in the long‐run but emphasized the sheer difficulty of making an anxious child go to school. Episodes were described of bedtime worries and sleepless nights escalating to a peak of physical and emotional distress in the morning before school. A sense of helplessness was apparent in parents' descriptions of not knowing what else to do apart from keeping their child at home. Most parents also felt that school staff tended to underestimate the severity of distress demonstrated by their children.

Do we physically force her in? I don't know what the right thing was to do. Sometimes we were on the brink of physically forcing her and I don't know, that felt emotionally wrong to do that. When she's not being naughty, she is obviously incredibly scared and that doesn't feel right to force it, in those circumstances.

Parent interview.

The school refuses to be more helpful. They seem to treat it as an inconvenience and it's not. It's a very visceral thing that the children are going through. Watching his expression and watching his emotions on those mornings, it's not fake, there's no way that a child could look so haunted or terrified as [child's name] does without it being a very real fear.

Parent interview.

School non‐attendance was perceived by the majority of professionals to be a matter of parental choice. Professionals were aware that children might find it difficult to get ready for school, but felt that parents needed to stand their ground.

It is mainly to do with parenting, I suppose. Sometimes what we talk about is ‘easy parenting,’ not wanting to challenge children when they don't want to go into school or having an easy day as a parent and not having that battle about coming in, just because it makes life easier.

Headteacher interview.

3 Areas for additional support

*Re‐building positive relationships with school staff* The deterioration of parent‐school relationships was a common experience. Parents explained that opportunities were urgently needed to discuss their child's difficulties openly and without judgment from school. A few parents also highlighted counterproductive communication strategies used by school staff, such as excessive reassurance that the child is ‘fine’ in school.

I was constantly in touch with school when she was in year 5 originally when it [SAPs] started, and they were actually making out that they understood… But [in year 6] there was just nothing that came back from the school from a supportive point of view of helping me get her to engage in it whatsoever.

Parent interview.

They were telling me he was fine, to almost prove that it wasn't their issue. Looking back now, I think they were doing it for reassurance, ‘He's happy here, you don't need to worry during the day.’ But it felt to me like the opposite. It felt to me like, ‘We're doing everything right, you're the one with the issue.’

Parent interview.

Conversely, professionals commonly recognized the importance of building positive relationships with parents and the need to rebuild these after COVID‐19 disruptions through proactive communication.

Where you [schools] have regular contact [with families] and then the family feels held and remembered … but it's difficult for schools to provide that level of resource.

Educational Psychologist interview.

*Early intervention* Some parents noted positive experiences of timely support directly from schools. This included having input from a dedicated staff member (e.g., Integrated Family Coach or School Nurse). However, the majority of parents reported that they had received no formal support, particularly during the emergent phase of their child's difficulties. The interviews further revealed that access to relevant external agencies (and particularly child mental health services) was restricted due to high thresholds and limited knowledge about how and where to seek help.

Every local resource [that] I've ever been pointed in the direction to, I don't meet the threshold for because it's not bad enough.

Parent interview.

Some professionals expressed concerns that COVID‐19 had worsened supply‐side barriers to early intervention.

This year has been a bit up and down, because normally I would meet with head teachers or people in primary schools every term, if not more often, depending on their level of need and talk about early intervention. So identifying students that you know were having a bit of a wobble before it becomes entrenched and then unable to actually go to school, because it's such a barrier getting them back once they stop going.

ESBAS officer interview.

*Peer support* Parents commonly expressed preferences for seeking advice from fellow parents with similar experiences, rather than consulting with school staff. It was felt that sharing experiences with peers can provide validation, emotional support and learning of practical solution‐oriented strategies. Some parents suggested that peer discussions could be facilitated in a helpful way by a trained person to ensure a productive experience and to avoid fuelling negativity towards school.

Drawing on other people's strategies that other people have used. Feeling not so alone and your child is not the only person that is struggling. You would feel more supported if you are talking to other people and sharing your experiences and struggles.

Parent interview.

Having someone oversee [a peer group meeting is important] because obviously you don't want to turn it into a school bashing group. You don't want to turn it in to a ‘don't send your children’ here group.

Parent interview.

Most professionals also endorsed the potential benefits of peer support for parents. They sympathized with the challenge of managing SAPs and particularly mentioned the loneliness that many parents feel.

I think having any kind of forum where it brings people together that have similar experiences, most parents, in my experience, find that enormously helpful. Because otherwise they feel very alone, they feel very judged, and they feel that no one is there to help.

School Mental Health & Emotional Wellbeing Adviser interview.

A concern was also raised about the difficulty of managing diverse viewpoints within peer support groups, with the suggestion that targeted and tailored 1:1 support may have particular value.

A lot of vulnerable families come with a lot of issues, and parents have all sorts of life experiences as well. I think that 1:1 is really helpful because it can be really tailored to their needs as a family.

Headteacher interview.

*Improving SEN provision* Parents of children with SEN felt that mainstream schools typically fail to understand how their child's SEN plays a role in anxiety towards attending school. These parents believed that further provision and training for SEN was needed. For example, one child had recently moved to a special needs school and found that specialist knowledge and understanding of their child's SEN dramatically improved their attendance by providing him with tailored support and removing the label of being the ‘naughty child.’ In contrast, professionals felt that school staff were generally well trained in this area, although they did report that COVID‐19 and limited funding had restricted their capacity for delivering supplemental specialized support.

That experience, that understanding on how to deal with situations, understanding sensory issues, to even know how classrooms are laid out, with people so close together. They even have a therapy dog in his classroom and he loves that dog and things like that. And understanding immediately that he is anxious, it's anxiety, it's not you are being bad, it's just that you are finding it difficult. And it's just changed the whole, it's changed his life, really.

Parent interview.

## DISCUSSION

This study drew on the perspectives of multiple stakeholders to develop a broad understanding of SAPs and associated service priorities in the context of the COVID‐19 pandemic. Our results were organized into three main thematic areas. First, participants described a number of contributing risk factors for SAPs, including exacerbations of previous difficulties with school, COVID‐specific anxiety, difficulties adapting to new rules and routines, and concerns about academic catch‐up. Second, participants described ways in which parents and professionals responded to SAPs, encompassing school and home collaboration, navigation of statutory contexts and parental accommodations. Third, participants made recommendations about priorities for additional support, which included building positive relationships with educational staff, early intervention, peer support for parents, and improving SEN provisions.

Our findings indicate that many of the etiological factors known to contribute to the onset and maintenance of SAPs before the pandemic (Heyne et al., 2019; Melvin et al., 2019) also appeared to operate in the context of COVID‐19. This includes child factors such as anxiety and SEN; parenting behaviours; and school factors such as communication with families, school environment, and academic pressures (Gubbels et al., 2019; Melvin et al., 2019). COVID‐19‐related restrictions (e.g., social distancing, bubbles, new rules, removal of services) exacerbated these risk factors for children with pre‐existing SAPs and also precipitated the emergence of new SAPs in some children. It has been suggested that the pandemic may have more persistent impacts on biological and psychological functioning for some children, and/or on the risks to which they are exposed, with cascading effects for their mental health and associated outcomes over the longer term (Sonuga‐Barke & Fearon, 2021). Thus, monitoring attendance, and intervening early where difficulties do arise, is of the utmost importance.

For children with pre‐existing SEN, the negative impact of the pandemic on school attendance may have been particularly marked. Prior to the pandemic, children with SEN were already more than twice as likely to be persistently absent from school than a child without SEN (Department for Education, 2022). Heightened rates of mental health problems (Preece & Howley, 2018), and school‐based factors such as the lack of adequate SEN provision (Havik et al., 2014; Humphrey & Lewis, 2008) are thought to, in part, explain the increased prevalence of SAPs in children with SEN. In the current study, parents and education professionals noted that anxiety around COVID‐19, the frequent changes to COVID‐related rules and safety regulations within schools, and the consequent disruption to children's routines and the provision of specialist resources were particularly difficult for children with SEN. These findings echo pre‐pandemic research and underscore the importance of recognizing that certain groups of children may have significant and ongoing support needs related to SAPs, even as we emerge from the pandemic (Waite et al., 2020).

While professionals were mostly sympathetic to the difficulties faced by parents in getting a distressed child to attend school, some described unhelpful parental behaviours in line with research on parental accommodations as a maintaining factor in child anxiety problems (Iniesta‐Sepúlveda et al., 2020; Lebowitz et al., 2014). Parental accommodations are a key target in evidence‐based cognitive‐behavioural treatment protocols for child anxiety problems (e.g., Byrne et al., 2021; Lebowitz et al., 2014). Yet many parents in the current study indicated that they had received little or no professional guidance about managing high levels of child anxiety, despite their appeals for such support. Moreover, a significant minority of parents felt blamed by schools for the persistence of SAPs.

These findings point to a need for compassionate and non‐judgmental support for parents to address their child's SAPs and for this support to be accessible when problems first emerge. Strategies could be informed by existing interventions, which integrate parent behaviour training on how to respond to children's distress and disruptive behaviours within a cognitive‐behavioural treatment framework (e.g., Cartwright‐Hatton et al., 2018; Lebowitz et al., 2014). Moreover, intervention development could usefully incorporate elements of peer support and potentially involve parents in intervention delivery roles (e.g., as facilitators of groups comprising fellow parents; Thomson et al., 2014). Peer‐led formats are also highly relevant in the context of restricted access to specialist services both before and during the pandemic (Crawley et al., 2020; Crenna‐Jennings & Hutchinson, 2020; Huang & Ougrin, 2021). Finally, building better relationships with professionals was identified as an important priority. Research has shown that interventions aimed specifically at improving parent‐school collaboration can improve school attendance and other academic outcomes (Smith et al., 2020).

### Strengths and limitations

Relatively limited research has examined SAPs in primary school children. The use of surveys followed by interviews meant we could gather perspectives from a larger number of stakeholders before focusing in greater depth on issues that were most pertinent to families during the COVID‐19 pandemic. Moreover, the use of multiple stakeholder groups highlighted areas of agreement and conflict, and identified opportunities to reconcile areas of difference (Dannow et al., 2018; Heyne et al., 2020).

Several limitations also warrant consideration. First, the parent sample consisted only of mothers. Future research should explore fathers' priorities and behaviours in respect of SAPs, so that these can be taken into account in intervention development. Although our sample showed reasonable variation with regard to SES, it included predominately White British families. Thus, we were unable to explore variation in the experiences of SAPs in families from different ethnic groups. Data collection was also focused on the UK education system. However, studies conducted in educational settings from the US and New Zealand have documented similar difficulties with school attendance in the context of the COVID‐19 pandemic (e.g., Briesch et al., 2021; Jeffs et al., 2021; Kalvin et al., 2021; Kroshus et al., 2020). Notably, this international research, like ours, has found heightened child and parental anxiety related to school attendance (Jeffs et al., 2021; Kroshus et al., 2020), greater difficulties accessing adequate SEN provisions (Kalvin et al., 2021), and inconsistent home‐school communication (Briesch et al., 2021). Thus, our findings and practice implications are unlikely to be unique to the UK education system.

## CONCLUSION

This study has provided in‐depth insights into stakeholders' experiences of SAPs in the context of the COVID‐19 pandemic. There is a need for new interventions that tackle SAPs and mitigate longer‐term impacts of the pandemic on child outcomes. Relevant interventions should be easily accessible and address a range of modifiable risk and protective factors within and outside family systems. This may include providing parents with non‐judgmental support and effective strategies for managing and responding to children's anxiety associated with school attendance, as well as working towards building effective relationships with school staff. Future research is needed to develop and evaluate such approaches, drawing on the lived experiences of diverse families to strengthen feasibility, acceptability and outcomes.

## AUTHOR CONTRIBUTIONS

**Brontë McDonald:** Conceptualization; data curation; formal analysis; investigation; methodology; project administration; writing – original draft. **Kathryn J. Lester:** Conceptualization; formal analysis; methodology; supervision; writing – review and editing. **Daniel Michelson:** Conceptualization; formal analysis; funding acquisition; methodology; supervision; writing – review and editing.

## CONFLICT OF INTEREST

All authors declare no conflict of interest.

## Supporting information


Appendix S1

## Data Availability

The detailed coding framework used in this study is available on request from the corresponding author. Participants did not provide permission to share raw data (i.e., unprocessed transcripts) outside of the core research team.
