# Supplementary material for: ‘She didn't know how to go back’: School attendance problems in the context of the COVID‐19 pandemic—A multiple stakeholder qualitative study with parents and professionals
Source: Br J Educ Psychol. 2022 Nov 7;93(1):386–401. doi: 10.1111/bjep.12562 (PMC10099830; doi:10.1111/bjep.12562)
Supplement: Supplementary file 1 — Appendix S1 [file BJEP-93-386-s001.docx]

**Phase 1: Survey open-ended, free-text questions**

***Parent questions.***

1. Please describe difficulties your child has experienced with school attendance during the Autumn term 2020?
2. Please describe any difficulties your child has experienced attending school before the Covid-19 pandemic. *(i.e. before March 2020).*
3. How have you felt, as a parent, about your child attending school during the pandemic?
4. What reasons do you think might explain your child’s difficulties attending school? *If relevant, please consider pandemic-related factors but also other factors too such as school, family or child-related reasons. These factors may have been apparent before the pandemic and/or emerged during the pandemic.*
5. What external help has been provided to your child or family to help support your child’s attendance at school? If relevant, please describe one or two specific things that have or haven’t been helpful. *Please consider formal support that has been provided by school, other external agencies and any more informal sources of support.*
6. What additional support do you think would be helpful for your child, family or other families when a child is experiencing difficulties with school attendance?
7. On March 8^th^ 2021, Schools were fully reopened for all pupils. Please describe any difficulties that you’ve faced in preparing your child(ren) to go back to school, and/or in maintaining their attendance since then.
8. Is there anything else you would like to add about your child’s challenges with school attendance and how they could be supported?

***Professional questions***

1. What difficulties do you think children and their families have faced in preparing to go back to school, and/or in maintaining their attendance following each period of national lockdown? *i.e. in Autumn term 2020 and Spring term 2021.*
2. A) In your experience, what groups of children have been most likely to have unauthorised absences following each phase of schools reopening? *i.e. in Autumn term 2020 and Spring term 2021. This could include particular child/family characteristics, age groups etc*
3. Please explain what you think the barriers are to school attendance for these groups of children.
4. Based on your experience e.g. in your school/local area, how do recent trends in school absenteeism *(i.e. in Autumn term 2020 and Spring term 2021)* compare with patterns of school absenteeism before the Covid-19 pandemic?
5. Please describe any examples of good practice that you have been involved with directly and/or observed from (a) your own employer; and (b) other schools/external agencies to support children and families affected by unauthorised school absenteeism in the context of the Covid-19 pandemic.
6. How do you think we can better support children and families experiencing difficulties with unauthorised school absenteeism now and in the aftermath of the Covid-19 pandemic?
7. Is there anything else you would like to add about your experiences relating to school absenteeism and how children could be supported?

**Phase 2: semi-structured interview topic guide**

***Parent topic guide***

**Preamble: Thank you for taking the time for this interview today. You have been given an information sheet before this interview, did you have any questions about this information before we start?**

**I would like to note that we are focusing on absences that are often labelled by schools as “unauthorised absences”.** These absences exclude mandated self-isolation and closure school bubbles, illness, medical appointments, religious observations and external exams and assessment.

1. Can you tell me a bit about the difficulties that your child has experienced attending school over the last academic year that may have led to “unauthorised absences”?

Prompt: How do any difficulties they are experiencing now compare to any attendance difficulties they may have experienced before the Covid-19 pandemic?

1. Excluding issues around mandated self-isolation and closure of school bubbles, can you tell be about the reasons that you think might explain your child’s absences that are considered “unauthorized”, since the start of the pandemic.

Prompt: How has the covid-19 pandemic affected your child and their school attendance?

Prompt: Is there anything outside of school that may be affecting your child’s attendance difficulties, could you tell me a little about that?

Prompt: Is this something your child has always struggled with? Do you have some ideas why?

1. To what extent do you think that emotional factors, such as worry or frustration, for example, have played a part in your child’s difficulties attending school? How has this impacted on their attendance?

Prompt: What, if any, worries or frustrations has your child had about attending school and how has this impacted on their attendance?

Prompt: What, if any, worries or frustration have you (or other family members) had about your child attending school and how has this impacted on their attendance?

1. How have your child’s difficulties attending school affected you and your family?

Prompt: How, if at all, have you had to change your own life or the life of other family members because of your child’s difficulties attending school?

1. Can you tell me a bit about how you respond and some of the things you might say or do if your child is reluctant or unwilling to attend school?

Prompt: What kinds of things have you said or done that have been helpful?

Prompt: What kinds of things have you said or done that have been less helpful?

1. How much do you think the way you interact with or respond to your child when they are experiencing difficulties attending school has been helpful or not helpful?
2. Can you tell me a bit about any support you and/or your child have received during this last year that has been aimed at helping your child’s difficulties with attending school?

Prompt: What, if any, support has your child’s school provided that has been helpful/unhelpful?

Prompt: What, if any, support have you received from other professionals that has been helpful/unhelpful?

Prompt: What, if any, extra support or guidance from schools or other professionals would you like to see to help your child, and to help you?

Prompt: What has been the role of informal support, for example from wider family, friends, other parents experiencing similar issues or via social media have you received? Has this been helpful/unhelpful?

1. Can you tell me about any support you are aware of that is available for you and your child even if you might not have used it?

Prompt: Where would you typically go to get guidance and support for your child’s difficulties attending school?

Prompt: Too what extent is it easy or difficult to find out where to go for guidance and support? How have you found accessing that support?

Prompt: How easy is to understand how all the different sources of support and guidance fit together?

In the first stage of our research, what came up consistently is that parents often feel alone in supporting their child with their school attendance difficulties and felt it might be helpful to be able to share and talk about their concerns informally with others.

1. Can you tell me a bit about how you have felt, as a parent, while your child has had difficulties with school attendance?

Prompt: How have you coped with your child’s difficulties?

1. How helpful would it be to connect with/learn from other families whose children also have difficulties with school attendance?

Prompt: What was it/What do you think it is...about speaking to and hearing from other parents experiencing similar difficulties that you think would be helpful?

Prompt: Can you see any negatives to having this kind of support available to families? Or any barriers that might discourage families from using this kind of support?

1. How could these opportunities be facilitated in a way that is feasible and acceptable? I.e. online, pre-recorded, in-person, groups/1:1?

Prompt: What should be provided alongside this opportunity to meet with other parents? should it involve emotional support? Instructional support or any resources?

Prompt: (For parents with SEN children). Would you find it more helpful to be with parents of SEN children or parents without SEN children? What would be the benefit of this?

To what extent should this type of support involve schools or other professionals?

Prompt: Who, if anyone, from schools or other professional organisations do you think could be involved?

Prompt: What do you think their role could be?

Prompt: What concerns or problems could you see about also involving schools or other professionals in this type of support?

1. Can you talk about what the communication and relationship between you and your child’s school has been like about your child’s school attendance difficulties?

Prompt: What in particular about the communication and relationship between you and your child’s school has been helpful?

Prompt: What in particular about the communication and relationship between you and your child’s school has been unhelpful?

1. What, if anything, could be done to improve the communication and relationship between you and your child’s school?

Prompt: How might this impact on your child’s school attendance difficulties?

1. Anything else important to mention that you don’t think we’ve covered?
2. **I**n the next stage of our research, we will be inviting families to collaborate with researcher and educational staff to develop support or an intervention. Would you be willing to be contacted about participating in this next stage of research?

Can you tell me more about that? Could you give me an example?

***Professional topic guide***

1. Can you tell me about your current role in supporting school attendance difficulties?
2. Putting aside issues around mandated self-isolation and closure of school bubbles, can you tell me about patterns of **unauthorised** absences affecting primary pupils that in your school/area since the start of the pandemic?

Prompt: What factors have been associated with unauthorised absences and how do these compare with drivers of school absenteeism prior to the pandemic?

1. Can you describe how parents/carers have responded to children’s difficulties attending school?

Prompt: What kinds of things have parents said or done that have been helpful?

Prompt: What kinds of things have parents said or done that have been less helpful?

1. *.* What is the connection, if any, between parental anxiety (e.g., about the pandemic or other issues) and children’s unauthorised school absences?

Prompt: how important is it to address parents’/carers’ own anxiety as a contributing factor in children’s unauthorised absences from schools?

1. Can you tell me about the support that is available to help children and families experiencing difficulties in attending school?

Prompt: What, if any, support have schools provided that has been helpful/unhelpful?

Prompt: What, if any, support from other professionals that has been helpful/unhelpful?

Prompt: What has been the role of informal support, for example from wider family, friends, other parents experiencing similar issues or via social media? Has this been helpful/unhelpful?

Prompt: Where would parents typically go to get guidance and support for their child’s difficulties attending school?

Prompt: Too what extent is it easy or difficult to find out where to go for guidance and support and how various services fit together?

1. What difference, if any, do you think it would make if parents had opportunities to connect with/learn from other families struggling with school attendance difficulties?

Prompt: What would be the benefits?

Prompt: Can you see any negatives to having this kind of support available to parents? Or any barriers that might discourage parents/carers from using this kind of support?

How would these opportunities be facilitated in a way that is feasible and acceptable? i.e online, pre-recorded, in-person, groups/1:1.

Prompt: What should be provided alongside this opportunity to meet with other parents? should it involve emotional support? Instructional support or any resources?

Prompt: (For parents with SEN children). Would it be more helpful for parents of SEN children or parents without SEN children? What would be the benefit of this?

1. To what extent should this type of support involve schools or other professionals?

Prompt: Who, if anyone, from schools or other professional organisations do you think could be involved?

Prompt: What do you think their role could be?

Prompt: What concerns or problems could you see about also involving schools or other professionals in this type of support?

1. Can you talk about how your school/schools in your area have communicated with parents/carers in relation to school attendance problems during the pandemic?

Prompt: What in particular about the communication and relationship between you and the parents /carers has been successful/unsuccessful?

Prompt: could you describe some example communications you have had with parents about their attendance difficulties?

1. Anything else important to mention that you don’t think we’ve covered?
2. In the next stage of our research, we will be inviting families and educational professionals to collaborate with researcher to developing support or an intervention. Would you be willing to be contacted about participating in this next stage of research?
